# Needs and Attitudes of Older Chronic Back Pain Patients towards a Wearable for Ultrasound Biofeedback during Stabilization Exercises: A Qualitative Analysis

**DOI:** 10.3390/ijerph20064927

**Published:** 2023-03-10

**Authors:** Luis Perotti, Oskar Stamm, Lisa Mesletzky, Susan Vorwerg, Marc Fournelle, Ursula Müller-Werdan

**Affiliations:** 1 Department of Geriatrics and Medical Gerontology, Charité—Universitätsmedizin Berlin, Corporate Member of Freie Universität Berlin and Humboldt-Universität zu Berlin, 13347 Berlin, Germany; 2 Department of Ultrasound, Fraunhofer Institute for Biomedical Engineering, 66280 Sulzbach, Germany

**Keywords:** older adults, biofeedback, chronic back pain, ultrasound imaging, wearable, segmental stabilization, physiotherapy, deep learning

## Abstract

Chronic back pain has a high prevalence, especially in older adults, and seriously affects sufferers’ quality of life. Segmental stabilization exercise (SSE) is often used during physiotherapy to enhance core stability. The execution of SSE requires the selective contraction of deep abdominal and back muscles. Motor learning can be supported using ultrasound imaging as visual biofeedback. ULTRAWEAR is a mobile ultrasound system that provides deep learning-based biofeedback on SSE execution, which is currently under development. We interviewed 15 older chronic back pain patients (CBPPs) to investigate their pain management behavior, experience with SSE, as well as their needs and requirements for ULTRAWEAR. We also gathered information about future-usage scenarios. CBPPs reported a high willingness to use the system as a feedback tool both in physiotherapeutic practices and at home. The automated detection and evaluation of muscle contraction states was highlighted as a major benefit of the system compared to the more subjective feedback provided by traditional methods such as palpation. The system to be developed was perceived as a helpful solution to support learning about SSE.

## 1. Introduction

Chronic back pain (CBP, back pain lasting three months or longer and occurring almost daily [1]) has a high prevalence in Germany, and it is especially high in older adults. In 2020, the 12-month prevalence of back pain in general was 66% in women and 56.4% in men. The 12-month prevalence of chronic back pain among people aged 70 years and older was 28.0% in women and 17.4% in men [2]. From an international perspective, back pain is the most common musculoskeletal health problem in people over 75 years of age, increasing expenses for healthcare systems [3]. Chronic low back pain (LBP) prevalence in older adults is significantly higher than in younger adults. Furthermore, older adults with chronic LBP experience more significant effects and a longer duration of symptoms. Nonetheless, the rate of chronic LBP patients care seeking in older adults is lower than in younger adults [4]. 

Physiotherapeutic treatments show a moderate certainty of clinical evidence for effective treatment of chronic back pain [5]. However, not all of the treatment options for younger adults can be applied to older patients, as other comorbidities, such as osteoporosis, tumors, spinal infection, lumbar spinal stenosis, and dementia may limit their applicability [6,7]. Physiotherapists should adapt their treatments regarding senescent body changes that may make older adults vulnerable to persistent pain and declining physical activity and function [8]. 

Core stabilization exercises are a standard procedure in most physiotherapeutic pain treatments, and the benefits for low back pain patients are well documented in the literature [9,10,11,12,13]. Core stabilization exercises have a positive effect on pain, proprioception, functional disability, fear of movement, and balance [11,14], as well as overall quality of life [15]. The most common form of core stabilization exercises is segmental stabilization exercise (SSE) [16]. However, performing SSE is challenging for both the physiotherapist and patient. Therapists need to be properly trained to instruct these exercises, and patients with poor body awareness often have difficulty activating deep abdominal and back muscles and maintaining the contraction [17]. Most patients must first learn the correct contraction of the muscles. In SSE, the transverse abdominis (TrA) and lumbar multifidus (LM) muscles are particularly important. The lateral abdominal muscles TrA, internal oblique (IO), external oblique (EO), and LM control movement and provide stability to the trunk and spine [3]. Muscle contraction is often delayed and attenuated in patients with CLB [3]. Visual video feedback has proven to be beneficial for learning physical exercise execution and is superior to verbal feedback only [18]. The physiotherapist typically judges a successful isolated contraction of the TrA by observing the increase in the TrA thickness compared to that of the IO and EO. The relative thickness of the muscles can be measured using ultrasound [19].

Rehabilitative ultrasound imaging (RUSI) is often used by physiotherapists to assess and evaluate muscle morphology and contraction in relaxed conditions and during exercises [20]. The reliability and validity of RUSI is well documented in the literature. Some studies suggest that RUSI is even more beneficial in assessing muscle conditions compared to other techniques such as magnetic resonance imaging and electromyographic biofeedback [3,21,22]. Lee et al. [23] also described RUSI as the preferred and most effective method for delivering feedback during TrA training, compared to pressure-based biofeedback units and manual contact with voice commands. RUSI is also used to quantify the contraction of deep abdominal and back muscles, i.e., to measure the thickness and performance of the muscles in different conditions [3,24].

Intervention studies in which patients were given feedback on LM and TrA contractions via ultrasound images showed favorable results compared to a control group without visual feedback. RUSI, as a means of visual biofeedback, was found to improve continuous contraction and improve the ability to activate the LM and TrA muscles. The visual feedback helped patients learn correct activation; thus, fewer attempts were needed to achieve correct exercise execution [25,26]. However, these studies involved healthy and younger participants. One difficulty of RUSI is that ultrasound images of the deep abdominals and back muscles are difficult to interpret. Therefore, experienced and highly specialized professionals are required to safely interpret the images and avoid low intra-observer reliability [27]. 

Currently, approaches are being investigated in which muscles are automatically detected in ultrasound imaging via deep learning. Furthermore, the thickness of muscles in contraction and at rest is being measured automatically within the ultrasound image in real time. Such newly developed systems are detecting muscle tissue with almost as much precision as professional human operators [27,28]. However, these approaches only work for high-end stationary ultrasound systems with very good image quality. This makes monitoring of the musculature during dynamic exercise infeasible. Meanwhile, in other approaches, mobile wireless ultrasound systems are used to monitor the contraction states of a specific musculature [29]. Huang et al. [30] developed a mobile ultrasound system that uses ultrasound imaging to provide feedback on the contraction of the pectoralis major muscle in healthy younger subjects. Although wireless systems offer some mobility during exercises, no currently available mobile system can be used for deep abdominal and back muscles while analyzing and evaluating muscle location and contraction states via deep learning at the same time. In addition, there are also no deep learning solutions whose feedback is specifically intended for non-professionals or patients. Despite the high prevalence of chronic back pain in older adults, much of the current research in this area mainly addresses the middle-aged segment of the population [4]. Older people and their specific needs and usage requirements are not sufficiently considered in recent technology developments. This target group is usually hardly included in development processes or may only be included at a late stage. There are frequent arguments for strengthening the position of older adults as a target group in the research and development of technologies in order to design appropriate solutions [31].

### 1.1. Conceptual Framework

Against this background, the goal of the ULTRAWEAR project is the development of a portable wireless device for RUSI of the deep abdominal and back muscles during SSE. The future system should provide biofeedback on the correct performance of SSE. The system will use a deep learning approach to recognize muscular structures in the ultrasound image and their state of contraction in real time. To assure accessibility, the ULTRAWEAR system will be designed as low-cost hardware, consisting of a small ultrasound transducer and a mobile preprocessing electronics unit with wireless connection to a consumer electronics device, such as a tablet, for displaying the data and guiding patients through the SSE. The system under development is intended for use in the setting of physiotherapy practices and rehabilitation centers. This system will provide visual feedback on muscle contraction status during SSE.

In a prior study within the project, we collected expert requirements and attitudes regarding the system to be developed. We conducted single interviews with physiotherapists and medical physicians with extensive experience in the use of imaging ultrasound. The results were the foundation for the future ULTRAWEAR system, which will be discussed in the present study [32].

### 1.2. Research Questions

The present study aims to explore how the future ULTRAWEAR system can be adapted to the needs and expectations of the target group of older adults with chronic back pain. For this purpose, the experiences of older adults in dealing with chronic back pain and SSE, as well as ultrasound imaging in physiotherapy, were recorded. Furthermore, key elements and concepts of the system in development are discussed with the target group of chronic back pain patients (CBPPs). From this, requirements for the further development of the system were derived. Thus, the following research questions arose:

- What are the attitudes of older CBPPs towards using a mobile ultrasound device to obtain biofeedback when performing SSE?
- What needs do older CBPPs have for using a mobile ultrasound device to obtain biofeedback when performing SSE?
- How do older CBPPs evaluate the usefulness of a mobile ultrasound device for obtaining biofeedback during the performance of SSE?
- What are the requirements of CBPPs regarding the usage scenario of a mobile ultrasound device for biofeedback during the performance of SSE?

## 2. Materials and Methods

### 2.1. Study Design 

The conducted study had an explorative, qualitative approach. In addition, we used quantitative assessments to gather baseline data from the CBPPs. We collected qualitative data by conducting in-depth interviews and used the Consolidated Criteria for Reporting Qualitative Research (COREQ) guide for reporting. A systematic qualitative content analysis according to Mayring was applied for data analysis [33]. Ethical approval for the study was gained from the Ethics Committee of the Charité–Universitätsmedizin Berlin (No. EA4/182/21). The trial was registered on the German Clinical Trials Register (DRKS-ID: DRKS00026684; UTN: U1111-1271-0066).

### 2.2. Procedure 

#### 2.2.1. Screening

CBPPs were informed about the study via e-mail using the internal database of the Geriatrics Research Group of Charité–Universitätsmedizin Berlin, which contained a dataset of CBPPs from previous studies. For interested patients, a telephone screening was conducted to determine their suitability for the study. 

The following inclusion criteria were applied:

- Chronic back pain lasting longer than 6 months;
- 65 years of age or older;
- Independent mobility (mobility without help of others).

The exclusion criteria were as follows:

- Cognitive impairment;
- Sensory and/or motor deficits;
- Inability to actively perform exercises (especially while lying on the back);
- Malignant diseases and tumors of the spine, or fibromyalgia.

#### 2.2.2. Pilot Test

We conducted one pilot test before the start of the study. One 65-year-old woman without chronic back pain was interviewed using our semi-structured interview guideline and filled out the assessments. The pilot test lasted for two hours. The aim was to check the organizational process, as well as the comprehensibility of the questionnaires and the stringency of the questions. The pilot test participant was asked to point out any aspects that were difficult to understand and to suggest improvements. The observations from the interviewers were discussed after the interviews to improve the materials and the organizational process. The pilot test led to a change in the order of the questions, and some questions were removed from the interview guide. The final interview guide used for this study can be found in Appendix A.

#### 2.2.3. Study Procedure

The study was conducted in November and December 2021 in the facility of the Geriatrics Research Group at Charité–Universitätsmedizin Berlin. The interview lasted up to two hours per participant (*n* = 15). Three interviewers were involved in total. The interviewers were research associates of the Geriatrics Research Group: I1 was a male scientist with experience in public health and conducted six interviews; I2 was a male scientist, who is an experienced physiotherapist, who conducted four interviews and fulfilled the role of a physiotherapist in the study; I3 was a female student assistant with experience in human factors and design thinking and conducted five interviews. The study setting involved one interviewer and one protocolist (study staff) at each interview.

The procedure and aim of the study was explained to the subjects. After signing an informed consent form, participants completed questionnaires to collect data on general characteristics of the participants. The questionnaires comprised the following validated assessments: Affinity for Technology Interaction Short Scale [34]; and the Exercise Adherence Rating Scale [35].

**Figure 1:**
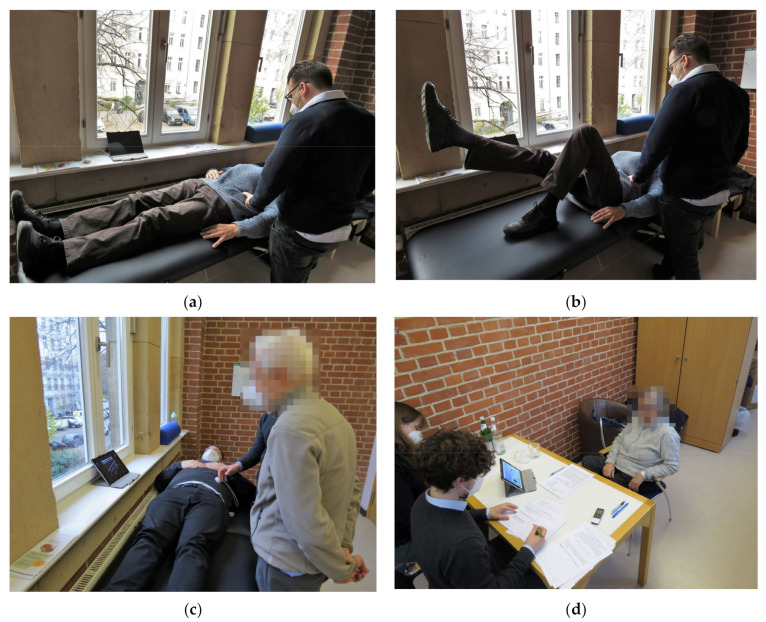
(**a**) Guided contraction of the deep abdominal muscles with palpation in supine position without visual feedback (demonstration; actual palpation was not performed on the clothing, but the skin surface); (**b**) guided contraction of the deep abdominal muscles with palpation in supine position with extended leg without visual feedback (demonstration; actual palpation was not performed on the clothing, but the skin surface); (**c**) demonstration of visual feedback (on a tablet) using an ULTRAWEAR mockup as a Wizard-of-Oz method; (**d**) overview of the setting during the qualitative interviews.

Subsequently, the first interview segment was about the participants’ experience with pain and segmental stabilization, if any. To ensure that all subjects had a basic understanding of the concept of segmental stabilization, a practice demonstration was performed under physiotherapeutic supervision. During all exercises, the participants laid in a supine position on a treatment couch and were asked to contract their lower abdomen and imagine pulling their belly button slightly inward towards the spine. This exercise is called an abdominal drawing-in maneuver [36] and is an exercise aimed to facilitate isolated contraction of the TrA. The three exercises were perception of breathing while performing a self-palpation of the abdomen, palpation of the TrA medially of the iliac crest with bent legs (Figure 1a), and the same while extending one leg (Figure 1b). 

This introduction into SSE was followed by interview segment two on the perception of segmental stabilization. In order for the participants to gain an understanding of a potential portable ultrasound system, a mockup system (consisting of an empty housing of an ultrasound transducer and a tablet) was used, and we followed the Wizard-of-Oz method [37,38]. One member of the study staff laid on the treatment couch, while one interviewer held the mockup transducer against his or her belly. A prerecorded ultrasound video was shown as an exemplary real-time ultrasound image. The muscles on the video were shown and their names, location, and function were explained by the interviewer (Figure 1c). 

In interview segment three, the subjects were asked about the usefulness of such a system and the requirements for the hardware and usage scenario were collected (Figure 1d). 

### 2.3. Materials

#### 2.3.1. Affinity for Technology Interaction, German Version

The Affinity for Technology Interaction (ATI) scale is a self-report measure, which assesses users’ ability to cope with technology [34]. The ATI is a nine-item scale, which uses a 6-point Likert scale ranging from “completely disagree” (1) to “completely agree” (6). The responses for Items 3, 6, and 8 needed to be reversed. The total score of the ATI scale was calculated as a mean across all nine scales and ranges from 1.0 to 6.0. A higher score indicates a higher user ability to cope with technology. The S5-strict quota sample (*n* = 232) was the sample with the oldest respondents (Age M = 48.81; SD = 18.57) in the validation paper by Fanke et al. [39], and this was used as reference sample for this study (Cronbach’s α = 0.87). 

#### 2.3.2. Exercise Adherence Rating Scale

We translated the Exercise Adherence Rating Scale (EARS) [35], which assesses adherence to prescribed home exercise. The translation was performed independently by two researchers and discussed as well as finalized after back-translation. The EARS is a 6-item questionnaire with a 5-point Likert scale ranging from “completely agree” (0) to “completely disagree” (4). The positively phrased items (items 1, 4, and 6) had to be reversed. A higher total score represents higher overall adherence. The EARS total score ranged from 0 to 24. The EARS scale was established with an internal consistency of 0.81, a test–retest reliability of 0.94, and construct validity of 70% [35,40]. 

#### 2.3.3. Mockup

**Figure 2:**
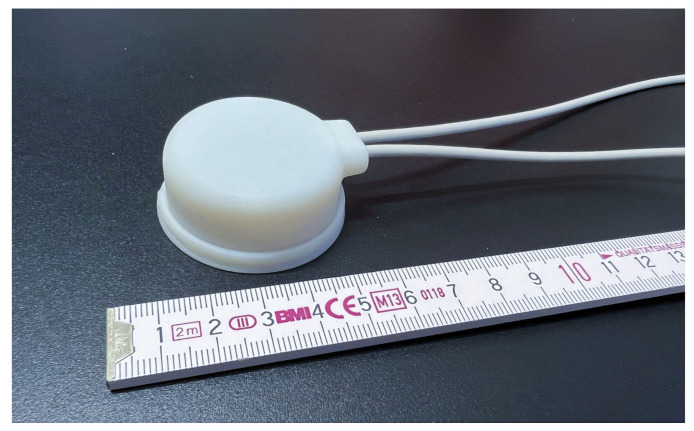
3D-printed ultrasound transducer mockup used in this study.

In order to explain the concept of the future ultrasound wearable ULTRAWEAR to the participants, we used a mockup in the form of a 3D-printed ultrasound transducer dummy (Figure 2) which was created by Fraunhofer IBMT. This was demonstrated to the participants in conjunction with an exemplary ultrasound image of the muscles on a tablet. The prerecorded exemplary video of the ultrasound image of the abdominal area (with the EO, IO, and TrA) was shown on the tablet to create the impression that a real-time ultrasound image was being visualized while the mockup was positioned on the abdominal region of study staff.

### 2.4. Participant Selection

Consecutive sampling was conducted in our study. CBPPs were initially approached via e-mail and were screened via telephone. We contacted 67 potential participants by e-mail and screened 22 of the 67 prospects via telephone. Of the 22 interested older adults, seven declined to participate. Of these, two refused without giving a reason, and two reported a fall beforehand, which prevented them from traveling to the study center. One person did not come to the appointment and one canceled due to illness. One interested person fulfilled the exclusion criterion of cognitive impairment and was excluded. After conducting 15 interviews, initial theoretical saturation occurred, which ended recruitment. At that point, we reached a code saturation, as we did not obtain any new themes or new codes. The pilot test was performed with a healthy 65-year-old woman. 

### 2.5. Data Collection and Analysis

The interviews were audio recorded with the participants’ consent. The audio files of all interviews were transcribed with the transcription software f4transkript (by dr. dresing & pehl GmbH, Marburg) according to predefined transcription rules. The content analysis was performed via systematic content analysis according to Mayring [33] with Atlas.ti 8 (version 8, ATLAS.ti Scientific Software Development GmbH, Berlin, Germany). We applied the quantitative data descriptive and inductive statistical methods using IBM SPSS Statistics 27 (Version 27.0, by IBM Corp. Armonk, NY, USA). For the purposes of inductive statistics, the data were examined for normal distribution using the Shapiro–Wilk test. We used an independent *t*-test to compare the differences in sex in the measures ATI and EARS. Researchers I1 and I2 analyzed the data. During this process, the audio material was analyzed according to certain rules and a coding agenda according to Mayring [33]. Initially, the first version of the coding agenda was created using theoretical considerations and the interview guide (deductive procedure). Next, each of the interviewers (I1–I3) independently coded the interviews of the pilot test and formed a categorization system using definitions from the material (inductive procedure). Finally, the three category systems were discussed, and a final coding manual was developed with categories, definitions, and examples. From the analysis of the interviews, 22 codes and 6 code groups could be identified based on content criteria. A total of 608 quotations were coded within the 15 interviews. Inter-rater reliability between the two analyzing researchers (I1 and I2) was calculated using Krippendorff’s cu-alpha for the semantic domains and Cu-alpha for all semantic domains. Krippendorff advises a reliance on variables with reliabilities above α = 0.800 and targeted minimum value with reliabilities no lower than α = 0.667 [41,42].

## 3. Results

### 3.1. Research Participant Characteristics

The main study was conducted with 15 older adults with CBP who met the inclusion criteria, and all signed informed consent after being given the opportunity to ask questions about the study. Table 1 shows the sample characteristics in detail. Eight of the participants were female (53.3%) and seven were male (46.7%). The total mean age was 75.7 years. All participants had LBP in the lumbar spine, most of them in combination with other areas.

#### 3.1.1. Exercise Adherence

For the Exercise Adherence Rating Scale (EARS) [35] the mean score of the participants in our study was 15.33 (SD = 5.51, *n* = 15). As a reference sample, the sample obtained by de Lira et al. [46] with the Brazilian Portuguese version (EARS-Br) was used. That reference study also included participants with non-specific chronic LBP, but with an age ranging from 18 to 60 years (mean age = 46.62, SD = 9.98). The participants in the study by de Lira et al. reached a slightly higher mean EARS score (M = 18.75, SD = 6.56, *n* = 108) than our sample. In our study, there was no significant difference between male and female participants (t(13) = 0.87, *p* = 0.200). However, female participants (M_female_ = 16.50, SD_female_ = 5.73) attained higher scores than male participants (M_male_ = 14.00, SD_male_ = 5.35).

#### 3.1.2. Affinity for Technology Interaction

For the Affinity for Technology Interaction (ATI) Scale, the following scores were obtained in our sample: M = 3.50 (SD = 1.07, Cronbach’s α = 0.82, *n* = 15). The reference sample S5-strict [39] avoided oversampling by matching the distribution of age, gender, and educational level in the population and showed similar results (M = 3.61, SD = 1.08, Cronbach’s α = 0.87). There was no significant difference for sex in the ATI total score in our sample, t(13) = 0.87, *p* = 0.079. However, men (M_male_ = 3.92, SD_male_ = 1.40, *n* = 7) had higher ATI scores than women (M_female_ = 3.13, SD_female_ = 0.73, *n* = 8). The reference sample S5-strict had similar results with it large sample (*n* = 232). Franke reported the following composition within the S5 sample: M_male_ = 4.13, SD_male_ = 0.94, *n* = 113; and M_female_ = 3.13, SD_female_ = 0.98, *n* = 119 (t(230) = 7.89, *p* < 0.001).

The following table (Table 2) lists the code groups used in the analysis of the qualitative interviews including the calculated Krippendorff’s cu-α [41,42]. As seen in the table, the calculated intercoder reliability was above 0.800 in most code groups and even above 0.900 in one code group. In the two code groups *“Prior experience with SSE”* and *“Usefulness and usage scenario of the future system”,* the value was below 0.800 but above the threshold of 0.667. This could have been due to the very broad category in the code group *“Usefulness and usage scenario of the future system”* and the low number of citations in the code group *“Prior experience with SSE”*.

### 3.2. Chronic Back Pain Management

The first set of interview questions concerned patients’ experiences handling chronic back pain. The questions referred to existing restrictions due to back pain during sports activities or in everyday life, and the interventions used to relieve the back pain. In addition, the interviewees were invited to comment on any existing experience with stabilization exercises and ultrasound.

Of the 15 interviewees, 11 reported various limitations in sports activities. These included exercises such as bridging, sit-ups, balance exercises, or stretching exercises. In addition, the interviewees also expressed discomfort when changing position or in specific positions. For example, when turning from one side to the other or when standing up from the supine position. In addition, certain exercise positions, such as sitting or standing, were described as difficult. 

In addition to restrictions in sports activities, eight interviewees mentioned limitations in everyday life. These mainly referred to changes in position, such as getting out of bed, turning in bed at night, and starting to walk. However, certain postures, such as sitting for a long time, standing or lying down, also caused restrictions in everyday life.

In the question about interventions to relieve back pain, all respondents expressed their opinion about taking painkillers. Six people vehemently rejected the idea of taking painkillers. Four participants regularly take painkillers, while six participants reported additionally or exclusively taking painkillers during acute pain conditions. Most interviewees (*n* = 13) already had experience with physiotherapeutic or osteopathic treatment methods for back pain. These included exercise therapy, spinal or aqua gymnastics, and methods such as massage, manual therapy, relaxation methods, heat applications, and acupuncture. Five participants had already participated in a rehabilitation program. However, two interviewees also received psychological support. The main self-help intervention mentioned was the performance of gymnastic exercises and stretching exercises. However, other forms of exercise such as walking, swimming, cycling, Pilates, and line dancing were mentioned. Passive interventions such as heat rubs, self-massages, and current applications were also indicated.

### 3.3. Prior Experience with Segmental Stabilization Exercises

Regarding the question about experiences with stabilization exercises, 11 of the 15 participants stated that they had no knowledge about the activation of deep muscles (*“No. At least not that anyone told me that it should help in that direction. The physiotherapist says certain exercises, but she doesn’t always say exactly which muscles are to be used*” P04, m, 69 y). However, through specific inquiries by the researchers, it turned out that 14 interviewees already knew exercises for stabilizing the deep trunk musculature, although they could not identify them as such. These experiences were formed, for example, in rehabilitation sports, in the gymnastics group, during yoga, in sling training, in Pilates, or in physiotherapy. Only one person had no experience of these experiences. In addition, the interviewees stated that the stabilization exercises require concentration and are exhausting but are also helpful and effective.

### 3.4. Prior Experience with Ultrasound in Physiotherapy

None of the 15 participants reported prior experience in the utilization of ultrasound imaging in physiotherapy when asked. Four of the CBPP reported having experienced ultrasound imaging during previous medical examinations (*“No, so I know ultrasound from medical examinations, when the kidney is examined for example. From there I know it, also from gynecological examinations, but not other than that.*” P03, f, 80 y). Two other subjects had prior experience in the use of ultrasound therapy as a pain treatment *(“Ultrasound yes, but not to visualize the deep abdominal and back muscles, but ultrasound treatment, shoulder and lumbar spine.*” P13, m, 77 y).

### 3.5. Feedback on Learning of Stabilization Exercises

After performing SSE as instructed by the study personnel, we asked the study participants about how they perceived the exercises they just completed. Of the 15 participants, 10 expressed that they found SSE in the supine position enjoyable and easy to perform. They indicated that they had no difficulty understanding or carrying out the exercise’s objective and manner of execution. The targeted contraction of the deep abdominal muscles was described as something that *“can be felt and understood”* (P09, m, 78 y and P12, f, 69 y). The remaining five participants, on the other hand, expressed that they found the exercises difficult, although some of them had prior experience with SSE. They perceived the SSE as challenging because, in particular, sensing the contraction of the deep muscles required a lot of concentration or could not be achieved at all. One subject emphasized that the exercise itself was not strenuous or complex, but that self-control of the exercise performance was not possible despite the self-palpation (*“I think I couldn’t feel it that well. So I’m not sure if it really activated my deep abdominal muscles.*” P03, f, 80 y). Some participants also struggled to contract their abdominal muscles without holding their breath in parallel. 

### 3.6. Attitudes towards the Future System

#### 3.6.1. Positive Attitudes towards the Future System

After the presentation, all of the CBPPs interviewed expressed a positive attitude towards the mockup of the ultrasound system shown. The goal was to determine factors influencing the acceptance of the system in development. Eight of the respondents perceived the system as *“interesting”*, *“impressive”*, and *“fascinating”*. The fact that the system automatically monitors the execution of the SSE and provides feedback on the quality of the exercise execution was positively highlighted by 10 subjects. In particular, the AI-assisted muscle and contraction detection was praised (*“That’s actually always the best thing. It’s independent of the person.”* P10, f, 74 y).

The importance of the ability to use AI to accurately measure muscle thickness and to make more specific statements about the level of muscle contraction was also highlighted.

“*So if it’s going to become part of the routine treatment, I’m convinced that it’s better to use artificial intelligence […] I’m convinced either the person using it [the physiotherapist] doesn’t recognize or doesn’t know exactly what’s really happening, doesn’t know the degree either, or doesn’t have time.”* (P06, f, 70 y)

Similarly, it was emphasized that the ultrasound image could provide new insights into the anatomy of the deep abdominal muscles for patients (*“Well, I’m learning something new. I have never seen myself like this before.*” P02, f, 73 y). Feedback originating from the technological system rather than from a human was also perceived as more motivational.

“*A person sometimes says, I’ve explained this to you x times, why aren’t you doing it yet? That gets you down. When a technical system says, listen, you’re on the right track, but try a little harder. Then, that’s OK. You don’t have an emotional relationship with the device, you’re just a signal generator, and that’s fine.”* (P05, m, 79 y)

#### 3.6.2. Concerns about the Future System

In addition to the positive aspects or expectations regarding the future use of the system in physiotherapy, we asked the older CBPPs about possible concerns they had regarding the hypothetical use of the ULTRAWEAR system in the future. Six of the subjects stated that they had no concerns about the potential system at all. The concern most frequently raised by the other respondents was the matter of privacy. Four of the respondents expressed that the system should gather only minimal data about the patient. Furthermore, the images and information captured by the device should only be stored locally on the device or should only reach the treating physiotherapist or close relatives (*“I would prefer my data to remain only on my own system, so that I can control who I want to give my data to. My physiotherapist could see them and my family.*” P04, f, 80 y). Other concerns included that the system could potentially be complicated to use and, therefore, could only be utilized in collaboration with a physiotherapist (*n* = 4). Thus, it was feared that patients could use the system incorrectly and might harm themselves (*“If the people who use it are honest with themselves and do it properly, then nothing happens. However, I figure there are always a few unreasonable people.”* P14, f, 77 y). In this context, one participant expressed that the system should only be used together with a physiotherapist (*“I would prefer doing it with a physiotherapist […] because I trust him. I could imagine it [the ULTRAWEAR system] making mistakes.”* P03, f, 80 y). One of the respondents was concerned that the use of the system could put physiotherapists’ jobs at risk, and another participant questioned the long-term purpose of the system. The participant said that if one could easily execute the SSE by oneself, after learning it with the ULTRAWEAR system, one could go on without the device.

### 3.7. Usefulness of the Future System

Ten of the participants stated that an important factor in the evaluation of the usefulness of the system was the fact that the analysis of the musculature was objectively carried out by a technical system. They stated that this contrasted with a more subjective evaluation by the physiotherapist and the perceived inaccuracy of palpation (*“Well, as I see it, it’s just not just the sense of touch, but I really see here what’s happening and what’s not happening.*” P04, m, 69 y). Thus, the assessment of the quality of an exercise execution was perceived as measurable by means of objectifiable parameters.

“*Yes, yes, it is quite important that there is an objective system behind it. That is precisely the advantage of what you are aiming for here, I think. There are more relaxed physiotherapists and then there are more exact ones who look closely and so on. And that is a very big subjective human factor. And what you are developing here has the advantage that you can objectify it.”* (P09, m, 78 y)

In this regard, the respondents had confidence that the system would make fewer errors than a human physiotherapist would (*“That’s what I think, the technology, it can’t actually lie.”* P10, f, 74 y) and that the use of the system would contribute to an overall increase in the exercise quality of SSE in patients due to its more accurate feedback. 

Another important aspect mentioned by six of the older adults was that it would be very useful to receive visual feedback on SSE. This was felt to be more engaging and easier to understand than, for example, feedback via palpation. Particularly for older adults with less experience in performing SSE, this would make it easier to learn the exercises. In addition, the older adults stated that patients would have the opportunity to receive immediate and simultaneous feedback on muscle contraction. One participant said that it is important to monitor the execution of the exercises.

“*The advantage is that you can really see how the muscles are moving. So before, when I did the exercise, they did show me that I can feel it. However, now it’s more convincing of course, I’m a more visual person.*” (P13, m, 77 y)

#### 3.7.1. Usefulness of the Future System in the Physiotherapy Setting

We asked the study participants whether they considered the potential use of the system in the physiotherapy practice, together with the physiotherapist, to be useful. Ten of the CBPP felt that application in the physiotherapy practice would be useful. It was particularly emphasized that the use of the ULTRAWEAR system would also provide additional helpful information for the therapist compared to classical assessment methods in SSE. This would allow the therapist to provide more specific feedback on exercise performance and to better supervise the exercises. Four of the respondents suggested that use of the system could reduce physiotherapists’ workload. In view of the growing number of patients with chronic back pain, the system could provide a way to address the growing time pressure in physiotherapy.

“*The ratio of suffering older adults to physiotherapists is changing, yes. There are more and more old people and back pain patients and in relation to that there are less and less physiotherapists, that’s why I think you developed the system.*” (P09, m, 78 y)

One respondent suggested that using the system, one therapist could supervise several patients at the same time. 

In contrast, three of the older adults doubted that the use of the potential system in physiotherapy practices could be useful. Two of them noted that using the system would require extra effort from the therapist and would strain resources within the already scarce treatment time. In particular, for experienced therapists who had worked with patients and SSE for a long time, the daily use of such a system in their own practice would be rather less useful.

“*Then, he [the physiotherapist] doesn’t have to be confronted with the new system. That is also an effort for the physiotherapist. If he has so and so many patients and has to apply the system, then he has extra work, that’s clear.*” (P05, m, 79 y)

In this context, however, it was also feared that the ULTRAWEAR system could endanger the jobs of physiotherapists in the future (*“If I could do it [SSE] alone, the system would take away jobs”* P11, f, 76 y).

#### 3.7.2. Usefulness of the Future System at Home

CBPPs were asked if, in addition to using the system in a physiotherapy practice, they could imagine using a system such as ULTRAWEAR independently in their own homes without the assistance of trained therapists. Eleven individuals stated that they would use the system on their own at home. An important factor mentioned by six of the CBPPs was the notion of using the system to perform SSE independent of location and time, while still receiving reliable feedback on the quality of the exercises. Thus, the presence of a physiotherapist to evaluate the exercises would be unnecessary. Furthermore, the use of the system could eliminate unnecessary travel times to physiotherapy practices and, thus, relieve patients. As a result, one participant said the system would allow patients to adjust their exercises according to their own preferences and daily schedule.

“*Yes, but in itself, you don’t even need the physiotherapist, I think. If you have mastered the exercises. You can do them even better than the physiotherapist or more often or at the right time. For example, if you do it lying down, in the morning after you get up. You can’t do that with a physiotherapist.*” (P09, m, 78 y)

However, a very important requirement mentioned by the older adults regarding the use of the system in one’s own home was that an introduction by a therapist or trained personnel should be given. This was expressed by 12 of the CBPPs, who noted that the introduction should include the main functions of the system, the correct positioning of the transducer on the body surface, and the interpretation of the results as shown by the system. This, they said, would be necessary to ensure safe and effective use of the system.

“*I can’t imagine that most people would get it right away […] there would have to be someone, at least in the initial phase. […] So there should be an initial control, that the person really does it right.*” (P05, m, 79 y)

Similarly, exercises performed at home should not be executed entirely free of therapists’ supervision. Two respondents noted that there should be regular contact with the therapist who, for example, examines the exercise data logged by the system, offers assistance, and advises on difficulties related to the exercises. In particular, the availability of exercise data gathered at home during sessions with the physiotherapist could motivate patients, and, thus, increase adherence to home exercises.

While one study participant considered the hypothetical use of ULTRAWEAR at home to be more helpful for people who had no prior experience with SSE, two other CBPPs rejected its use at home altogether. For these participants, such a usage scenario was unnecessary, since the exercises could be performed at home without the system. They feared that the use of the system would lead to less contact with the physiotherapist.

### 3.8. Requirements towards the Usage Scenario of the Future System

The older CBPPs were asked what additional factors they felt were important for the potential usage scenario of the ULTRAWEAR system in general, or what aspects they felt should be considered in the further development of the system. Seven of the participants noted that the visibility of the tablet, on which visual feedback on exercise performance is displayed, could be challenging while performing SSE in a supine position. If the tablet were to lie next to the exercising person, he or she would struggle to see the screen and exercise at the same time (*“Well, I lie on the ground while doing the exercises and have a low coffee table nearby at most. However, when it [the tablet] is positioned like that, I can’t see it from the floor.”* P06, f, 70 y).

In this regard, four of the respondents suggested that the biofeedback might be better displayed on a larger screen. They felt that visualization on a TV, which ideally could also be tilted, or even be mounted on the ceiling, would be more suitable for seniors, in order to be able to view the information while lying down (*“If it’s just for older people, […] then it [the screen] would have to be a bit bigger still. Or maybe you can even connect it to the TV or something.”* P09, m, 78 y). 

Another difficulty, according to four participants, could be that the division of attention during the exercises, on the execution of the SSE and the feedback from the system, could be very challenging for older adults. Simultaneous body awareness, correct exercise execution, and self-monitoring via the screen was described as very cognitively demanding. One subject suggested that some practice would be needed to be able to maintain this divided attention.

“*After all, you have to do two things at once, one is the physical, doing the exercises. And secondly, you have to visually pay attention to when to relax and contract the muscles. It’s probably a matter of habit and then it works automatically*.” (P05, m, 79 y)

With regard to the training itself, one respondent stated that SSE with the system should be conducted for a maximum of 30 min, otherwise the physical activity could be too exhausting.

## 4. Discussion

The older adults with chronic back pain interviewed in this study expressed a very positive overall reception to the underlying concept of the ultrasound system which will be developed based on their requirements. While most of the participants positively highlighted the possibility for deep learning-based muscle and contraction recognition, as well as the visual training feedback during SSE, they also mentioned the potential of the system to assist physiotherapists with exercise evaluation. The potential for improved feedback during exercises, as well as workload reduction, was positively highlighted in this context and was also addressed in other studies [47]. Many studies reported that during physiotherapeutic exercise programs, supervision of patients by the physiotherapist often plays a role in terms of adherence and training effect [48]. However, the participants of our study actually placed emphasis on the objective exercise evaluation and training feedback provided by the system, rather than the human connection to the therapist. Some participants even stated that use of the system without the therapist would be conceivable. This was also reflected by the high level of approval for the use of our future system in a home scenario by the older CBPPs. In our study, the participants reported a relatively high adherence to prescribed home exercises. However, most of the participants had little or no prior knowledge of SSE or deep abdominal muscle activation. Although researchers, such as Mannion et al. [49], have demonstrated that greater adherence to home exercises in CBPPs correlates positively with pain relief and disability, they did not consider the quality of exercise performance. The participants of our study expressed fear of incorrect exercise performance during unsupervised SSE. Some subjects mentioned that recording data on SSE performance at home and having it reviewed by the physiotherapist would further increase motivation to engage in home exercises. In self-management of the day-to-day implications of the chronic condition, many CBPPs wished to receive ongoing support and supervision by therapists after discharge from physiotherapy treatment [50]. The participants in our study estimated that the ULTRAWEAR system might offer a degree of supervision of SSE execution in home use, even outside of physiotherapy treatment.

In summary, a positive overall attitude towards an ultrasound wearable for feedback on SSE was evident in our study. Thus, there is a possibility that our upcoming system could be accepted by the target group in the future.

One advantage of our future system over stationary ultrasound systems is its wireless design, which allows for mobile use. This permits its use during exercises that are more dynamic and in different positions, which would not be feasible with a hardwired system. However, when considering exercises while using the ULTRAWEAR system, the needs and requirements of the target group of older adults should be carefully considered. In our survey, some subjects reported difficulties maintaining certain positions during exercise and in switching positions. This aspect should also be considered when choosing exercises to be performed with the ULTRAWEAR system. A possible implication for the proposed use of the system might be that at the beginning of a number of therapy sessions, only exercises that are performed while the patient is in a supine position should be executed. In the further progress of therapy, these exercises can be expanded by more complex exercises, depending on the subject’s individual conditions [51]. Waddell et al. [52] suggest that there should be steady, but realistic increments of exercise quotas.

The older adults who participated in our study had a positive view of the usefulness regarding our potential ultrasound system, especially in comparison to traditional feedback methods during SSE (such as palpation); ultrasound imaging as a biofeedback method was considered to be favorable in our survey. Visual feedback about muscle contraction state was suspected to be a more effective learning method. As demonstrated by existing studies in the area of RUSI in physiotherapy, engaging visual feedback can support learning of SSE [53], but has to be presented in a way that is suitable for the intended target group. 

Regarding the provision of multimodal pain therapy to older adults, von der Lippe et al. [2] demanded the improvement of health policy frameworks and increased consideration of the requirements of the target group. Especially for older adults, therapy programs would have to be specifically tailored towards their needs and reduce existing barriers, such as individual or public mobility restrictions. This, they said, is necessary to improve the care situation of older CBPPs. Therapy programs aimed towards older adults should also be made available as mobile treatment options for patients who have limited mobility. In a study by Franco et al. [54], older patients indicated that eliminating the time taken to travel to physiotherapy sessions would improve care provision. The use of ULTRAWEAR without a physiotherapist in the patient’s own home might have the potential to improve the care situation CBPPs encountering mobility problems and addressing the existing shortage of physiotherapists in various countries [55]. The CBPPs interviewed in our study could very well imagine the use of such an ultrasound system at home without continuous supervision. However, the respondents desired initial instruction by the physiotherapist. Likewise, the formulated fears of incorrect exercise performance due to a lack of supervision should also be considered. These aspects should be taken into account in the development of our system to ensure usefulness for our target group. At the same time, SSE should not be considered a sole therapy method, but as part of a comprehensive therapy plan for chronic pain [56]. Embedding SSE in a multimodal pain program should be considered in further evaluations of ULTRAWEAR. 

The older adults interviewed in our study expressed some requirements towards the future usage scenario of the ultrasound wearable. They regarded the tablet screen as too small for visibility during training. This was accompanied by issues regarding potential positioning of the device during SSE. In many stationary ultrasound devices, biofeedback during exercises is presented on a computer screen, which is usually built-in to the system. The screen can often be tilted or the image can be displayed on larger screens such as a TV screen [22]. Therefore, the possibility of reproducing the image on a larger device, such as a computer monitor or a TV, should be considered for the further development of ULTRAWEAR. Furthermore, the study participants criticized the positioning of the tablet, as well as the simultaneous execution of the exercise and the visual perception of the training feedback. One solution could be that the physiotherapist initially holds the tablet visibly for the patient during the first sessions. In the following sessions, he could use it only for himself as a means of control, and selectively for the patient to correct or discuss the exercises.

A study by Herbert et al. [57] compared the extent to which RUSI in deep-muscle contraction during SSE should be shown at variable times during exercise repetitions or continuously during exercise performance. The researchers found that in healthy subjects, variable feedback was preferable to constant visual feedback. Subjects in the training group with variable visual feedback had better retention of muscle recruitment after eight sessions. However, in this study, healthy and younger subjects were considered. Most older subjects with chronic back pain must initially be taught to overcome fear-avoidance beliefs in order to perform SSE [58]. The CBPPs interviewed in our survey had some prior experience with SSE, but likewise, difficulties performing SSE were reported by some patients. To facilitate the learning of the exercises, a possible training design could include continuous visual biofeedback during SSE throughout the first few sessions. In the following sessions, visual feedback could then be included as needed. This might make exercise execution more independent and effective after a couple of sessions. 

However, the effectiveness and sustainability of RUSI in SSE is not yet fully proven. The effect of RUSI on exercise performance is still being researched. Currently, several RCTs are being carried out to investigate the effect of core stability exercise using RUSI in non-specific lower-back-pain patients with a follow-up period of 6 months [59,60]. However, since the studies do not include detection and processing of the ultrasound images via a deep learning approach, do not use mobile ultrasound systems, and provide no specific preparation of the visual biofeedback for older adults, it is difficult to derive the effects as an implication for the future efficacy of the ULTRAWEAR system. Thus, based on the findings of this study, a prototype of an ultrasound-based wearable will be developed and examined in a future clinical study.

### Strength and Limitations

The strength of our study lies in the involvement of older adults in the development process of an innovative solution to improve the physiotherapeutic treatment of CBPPs. Older adults are often overlooked in the development process of new technological systems. The results are highly relevant to improve the physiotherapeutic care of patients with chronic back pain in the future.

However, certain limitations of the study should be considered when interpreting the results. On the one hand, we only used a mockup of the system in the demonstration of the system due to its early stage of development. Thus, the statements of the older adults may not concur with the user experience of the final system. Further technical development of ULTRAWEAR is still ongoing, meaning that ultrasound imaging of the final system could be qualitatively inferior to the images shown to the participants. In addition, we interviewed a relatively homogeneous group of older adults in our study, as evidenced by the relatively high interest in technical systems. When considering the results of the EARS questionnaire, it should be noted that it was an unofficial translation into German by the study personnel.

## 5. Conclusions

The CBPPs interviewed in this study expressed a very positive opinion towards the ultrasound-based wearable that used deep learning to provide visual biofeedback on SSE execution. Likewise, they indicated a high level of willingness to use the system as a supporting tool in the execution of SSE in the context of physiotherapeutic treatment. The respondents could imagine using the system in both of the scenarios presented to them (use in the physiotherapy practice and in their own home). In particular, the evaluation of exercise performance and exercise quality by the AI was highlighted as a major benefit of the system. The use was considered to be complementary to the more subjective human feedback given by the therapist. Confidence in the automated assessment of exercise execution was also evident in that feedback from the system was preferred by some respondents to feedback from the physiotherapist. This was attributed to the benefit of objective muscle contraction measurements and exercise feedback provided by the system. Similarly, the potential of the system in assisting physiotherapists with exercise evaluation was also mentioned by respondents. The interviewed older adults with chronic back pain perceived an ultrasound-based wearable as a beneficial tool for regular physiotherapeutic treatment as well as for home use. The concept of a wearable such as ours was perceived as a helpful tool supporting the learning of SSE, which might improve self-management of SSE in home exercises.

## Figures and Tables

**Table 1:** Study sample characteristics.

Sociodemographic Data	Sample
Sample size total [*n*] Sex (Female/Male)	15
	8/7
Age [M (SD)] Age range [min.–max.]	75.67 (5.04) 69–85
**Highest educational attainment [*n*]**	
University	6
Advanced technical college certificate	6
High school	1
Secondary school	1
Main school	1
**Marital status** single married divorced widowed cohabiting	1 10 0 4 0
Pain intensity-NRS [M (SD)] ^1^	4.40 (1.91)
Pain Enjoyment of Life and General Activity Scale-PEG [M (SD)] ^2^	3.98 (2.19)
**Associated diagnoses of chronification (most stated) [*n*]**	
Unspecified LBP	5
Disc herniation	3
Spinal stenosis	2
Scoliosis	2
**Comorbidities (most stated) [*n*]** Hypertension Osteoarthritis	3 2

^1^ Numeric Rating Scale [43,44]. ^2^ The Pain, Enjoyment of Life and General Activity Scale [45].

**Table 2:** Code groups used for the analysis and calculated intercoder reliability.

Code Group	Calculated Krippendorff’s cu-α
Chronic back pain management	0.850
Physical limitations due to chronic back pain	0.827
Prior experience with SSE	0.769
Learning of SSE exercises	0.846
Prior experience with ultrasound imaging	0.925
Attitudes towards the future system	0.893
Usefulness and usage scenario of the future system	0.710
Total score	0.809

## Data Availability

The data presented in this study are available on request from the corresponding author.

## Abbreviations

LBP	Lower-back pain
SSEs	Segmental stabilization exercises
TrA	Transverse abdominis muscle
LM	Lumbar multifidus muscle
IO	Internal abdominal oblique muscle
EO	External abdominal oblique muscle
RUSI	Rehabilitative ultrasound imaging
CBP	Chronic back pain
CBPP	Chronic back pain patients

## Appendix A

Qualitative interview guide used in this study

1. What measures do you use with regard to your back pain?
2. In therapy, deep muscle activation (e.g., abdominal muscles) is often taught to back pain patients.
  a. Did you have any prior experience with such stabilization exercises?
  b. Have you found the exercises to be effective?

3. How do your physical limitations or psychological barriers restrict you from exercising?

Performing SSE as instructed by the study personnel

4. Have you already experienced the exercise in this or a similar kind with a physiotherapist?
5. Did the exercises cause you any difficulties?
  a. If yes, what was the difficulty of this task?

6. Has ultrasound been used in one of your prior physiotherapy sessions to help you visualize the contraction of the deep abdominal muscles?
  a. If yes, how did you feel about that experience?

Demonstration of the ULTRAWEAR mockup

7. What is your first impression of the system?
8. What do you think about the use of such a system within physiotherapy treatment?
9. If you think about the exercises you performed earlier:
  a. Do you think ultrasound imaging would help you in learning how to achieve targeted contraction of your deep abdominal muscles?
  b. Do you think the demonstration changed your perception of your abdominal muscles?

10. Do you have any concerns about using ultrasound in your physiotherapy treatment?
11. Do you have any general concerns using the ULTRAWEAR system?

[Demonstration of the ULTRAWEAR UI]

[…] Questions 12 to 18 are not part of this paper and will be included in a separate publication regarding the UI of our system

19. Could you imagine using the system at home on your own without the supervision of a physiotherapist?
20. Do you have any further remarks you would like to share with us or issues you would like us to consider as we move forward with our project?

Thank you for your time and participation in our study.
